# Hospital-admitted COPD patients treated at home using telemedicine technology in The Virtual Hospital Trial: methods of a randomized effectiveness trial

**DOI:** 10.1186/1745-6215-14-280

**Published:** 2013-09-03

**Authors:** Anna Svarre Jakobsen, Lars C Laursen, Birte Østergaard, Susan Rydahl-Hansen, Klaus V Phanareth

**Affiliations:** 1 Research Unit of Clinical Nursing, Bispebjerg & Frederiksberg University Hospital, Bispebjerg Bakke 23a, DK 2400, Copenhagen, NV, Denmark; 2 Medical Department O, Herlev University Hospital, Herlev Ringvej 75, DK 2730, Herlev, Denmark; 3 Research Unit of Nursing, Institute of Clinical Research, University of Southern Denmark, Campusvej 55, DK 5230, Odense M, Denmark; 4 Medical Department M, Frederiksberg University Hospital, Nordre Fasanvej 57, DK 2000, Frederiksberg, Denmark

**Keywords:** Acute exacerbation, Telemedicine technology, COPD, Readmissions, Hospital admission, Telehealth

## Abstract

**Background:**

Recent reviews suggest that telemedicine solutions for patients with chronic obstructive pulmonary disease (COPD) may prevent hospital readmissions and emergency room visits and improve health-related quality of life. However, the studies are few and only involve COPD patients who are in a stable phase or in-patients who are ready for discharge. COPD patients hospitalized with an acute exacerbation may also benefit from telemedicine solutions. The overall aim is to investigate a telemedicine-based treatment solution for patients with acute exacerbation of COPD at home as compared to conventional hospital treatment measured according to first treatment failure, which is defined as readmission due to COPD within 30 days after discharge.

**Methods:**

COPD patients with acute exacerbation who fulfilled the eligibility criteria and were from two university hospitals in Denmark were randomized (1:1) by computer-generated tables that allocated treatments in blocks of four to receive either standard treatment at the hospital or the same standard treatment at home using telemedicine technology (that is, a video conference system with a touch screen and webcam and monitoring equipment (spirometer, thermometer, and pulse oximeter)). Patients treated in the telemedicine group were backed up by an organizational setting securing 24/7/365 online access to the hospital, as well as access to oxygen, nebulizer therapy, oral medical therapy and surveillance of vital parameters from home monitoring devices. Patients in both groups were discharged when clinically stable and when fulfilling five pre-specified discharge criteria. Follow-up was performed at 1, 3 and 6 months after discharge.

The primary outcome was treatment failure defined as readmission due to exacerbation in COPD within 30 days. Secondary outcomes were death from any cause, prescription of additional antibiotics or steroids, need of intubation or non-invasive ventilation, emergency room visits, visits to the general practitioner, lung function, bed days, health-related quality of life, healthcare costs and user satisfaction.

**Results:**

Enrollment of patients started in June 2010 and ended in December 2011. Follow-up ended in May 2012. Results were analyzed in 2013.

**Conclusions:**

The results may have implications on future hospital treatment modalities for patients with severe exacerbations in COPD where telemedicine may be used as an alternative to conventional admission.

**Trial registration:**

Clinical Trials NCT01155856

## Background

According to WHO (World Health Organization), chronic obstructive lung disease (COPD) will become the third leading cause of death worldwide in 2030 [1]. More than 430,000 people in Denmark are affected by COPD [2] - one of the highest prevalence’s in the world - and every year 23,000 hospital admissions are due to COPD [3]. Concomitantly with an aging population [4] the frequency of chronic disease is increasing and causing increases in healthcare costs which in turn has stimulated research and innovation in technology solutions [5].

Several initiatives supported by technology, among these telemedicine for patients with chronic disease, are tested to explore whether alternatives are, when used correctly, feasible and safe for the patient and cost-effective compared to conventional care [6].

Clinical evidence is a precondition for implementing telemedicine solutions into the clinical practice [7].

### Rationale

Recent reviews [7-10] with focus on telemedicine for COPD patients revealed very few randomized clinical trials (RCT’s), and the trials included in the reviews were small and multimodal with telemedicine as only part of the intervention, making it difficult to determine the effect of telemedicine alone. Furthermore the trials comprised data on newly discharged or stable COPD patients only.

The reviewers concluded that some telemedicine solutions for COPD patients may significantly reduce the number of hospital readmissions and emergency room visits [8,9] and may also have a positive effect on health-related quality of life [9]. A recent study examining the effect of telehealth on use of secondary care and mortality has found telehealth to be associated with lower emergency admission rates [6]. The study involved 3,230 patients with diabetes, chronic obstructive pulmonary disease, or heart failure who were recruited from practices in the UK. Evidence is, however, still poor and it is suggested that future trials involving telemedicine for COPD need to have improved designs and more rigorous attention to intervention specification and overall aim, to outcome definition, measurement and health-economic analyses [7]. It is also mentioned that there is no evidence that these interventions are embedded in routine clinical care [7]. In this article we describe the methods of The Virtual Hospital Trial (VHT) where we have endeavored to overcome aforementioned significant caveats in regard to design, intervention specification, outcome definitions etcetera, and, in addition, we describe how the intervention was embedded in the routine clinical care at the hospital.

### Purpose

The overall aim of this study was to investigate a telemedicine-based treatment solution for patients with acute exacerbation of COPD at home compared to conventional hospital treatment as measured by first treatment failure, which is defined as readmission due to COPD within 30 days after discharge. Use of additional medicine and healthcare services due to COPD, mortality rate, physiological parameters, and health-related quality of life, healthcare costs, adverse events and user satisfaction are also evaluated.

## Methods

### Design

This was a consecutive, randomized, open, parallel group, multicentre trial design. The study was planned as a PhD study with a duration of 3 years, starting with 18 months of enrollment (June 2010 to December 2011). All enrolled patients were followed for 6 months at follow-up visits 1, 3 and 6 months after discharge. Follow-up finished in May 2012.

### Study settings

Patients were recruited consecutively from the emergency departments at two University Hospitals in the area of Copenhagen, Denmark.

### Patients

#### ***Eligibility criteria***

The eligibility criteria are listed in Table 1. Patients who met the inclusion criteria and none of the exclusion criteria were consecutively enrolled in the trial. Staging of COPD was done according to the GOLD criteria, which is a global initiative and strategy for the diagnosis, management and prevention of COPD [11]. The severity grade was based on historical clinical data obtained through the patient records. Although the severity grade of COPD cannot be determined during an exacerbation, measurements were made prior to randomization to obtain baseline values and to confirm that the patients had a decreased, obstructive lung function in addition to a known diagnosis of COPD.

**Table 1:** Eligibility criteria

**Inclusion criteria**	**Exclusion criteria**
• COPD GOLD stage III-IV (that is, FEV1/FVC <0.70 and FEV1 <50% of predicted value).	• Need of NIV or manual ventilation at time of randomization
• Acute exacerbation of COPD (for example, Anthonisen *et al*. definition [12]).	• Severely overweight^b^
• >45 years	• Serious comorbidity:
• pH >7.35	• Unstable heart disease^c^
• Compliance (see, hear, the ability to understand and carry out self-monitoring tasks).	• Poorly regulated diabetes^d^
• Expected hospitalization > 2 days^a^	• Malignancy
	• Other diseases/conditions that make participation impossible (dementia, delirium etcetera).
	• Temperature >38°C or need for IV antibiotics
	• Noncompliance (nursing home resident, tourist etcetera).
	• Participation in another trial

^a^judged by the attending doctor after initial acute management.

^b^severely overweight, causing immobility or Pickwick Syndrome.

^c^angina pectoris, changes in the electrocardiogram that needed immediate treatment during admission.

^d^need for additional insulin during admission.

*COPD* chronic obstructive pulmonary disease, *GOLD* the global initiative for chronic obstructive lung disease, *FEV1* forced expiratory volume in 1 second, *FVC* forced vital capacity, *NIV* non-invasive ventilation, *IV*, intravenous.

### Randomization

Consenting patients, who fulfilled the inclusion criteria and none of the exclusion criteria, were consecutively randomized. An external center-used computer generated tables to allocate treatments in fixed blocks of four and patients were randomized 1:1 to either intervention or control group. Each hospital was provided with sequentially numbered, sealed and opaque envelopes from the external center in batches of ten envelopes so that the allocation sequence was concealed from the investigators assessing and enrolling participants. The sealed envelope was not opened by the patient until after the patient had signed a written consent form. The allocation concealment mechanism was monitored closely by the investigators to ensure that envelopes were never resealed and to ensure patients were entered correctly in the study no matter what allocation the envelope revealed.

### Blinding

Whereas patients and health personnel were not blinded after randomization, outcome assessors and data analysts will be blinded to the allocation, however for some of the secondary outcomes it is difficult for researchers to remain blind as the patients knew which group they were in.

### Outcomes

#### ***Primary outcome***

The primary outcome was treatment failure defined as:

Readmission due to an exacerbation in the chronic obstructive lung disease within 30 days.

Only readmissions occurring after being discharged from the telemonitoring group or the usual care group was designated a readmission. Patients in both groups were discharged when fulfilling five pre-specified discharge criteria and all telemonitoring equipment was removed from the patient’s home the same day of discharge.

#### ***Secondary outcomes***

The secondary outcomes were; 1) Prescription of additional antibiotics or steroids due to further exacerbation, 2) need of non-invasive ventilation (NIV) or mechanical ventilation, 3) death from any cause, 4) emergency room visits, 5) visits to the general practitioner, 6) lung function (FEV1, FVC, FEV1% predicted), 7) bed days, 8) health-related quality of life, 9) healthcare costs, 10) applicability, 11) user satisfaction, or 123) adverse events.

### Trial organization

One of the study doctors or a specially trained study nurse screened the electronic patient boards every morning in the emergency departments at each hospital for eligible patients. If any possible candidates had been admitted within the previous 24 hours the patients were informed in spoken and in written form about the study and were allowed one hour to decide whether they wanted to participate or not. Prior to randomization patients were treated according to the hospital’s protocol for exacerbations in COPD, which included a scoring algorithm to decide whether a patient was candidate to treatment with NIV or could continue care at the ward. The scoring system used measurements of blood gasses, oxygen saturation, the respiratory frequency and the level of consciousness to stratify the patients. In the acute phase (0 to 2 hours) all patients followed a predetermined regime monitoring arterial blood gasses, giving nebulized bronchodilator agents, oxygen therapy, corticosteroids and antibiotics in fixed doses by the attending doctor. After the acute phase it was determined through the scoring algorithm whether the patient needed intensive care (NIV/manual ventilation), continued care at a hospital ward, or could be discharged from the hospital. The patients who needed continued care at the ward were thereafter asked to participate in the trial.

### Interventions

#### ***Experimental group***

**Figure 1:**
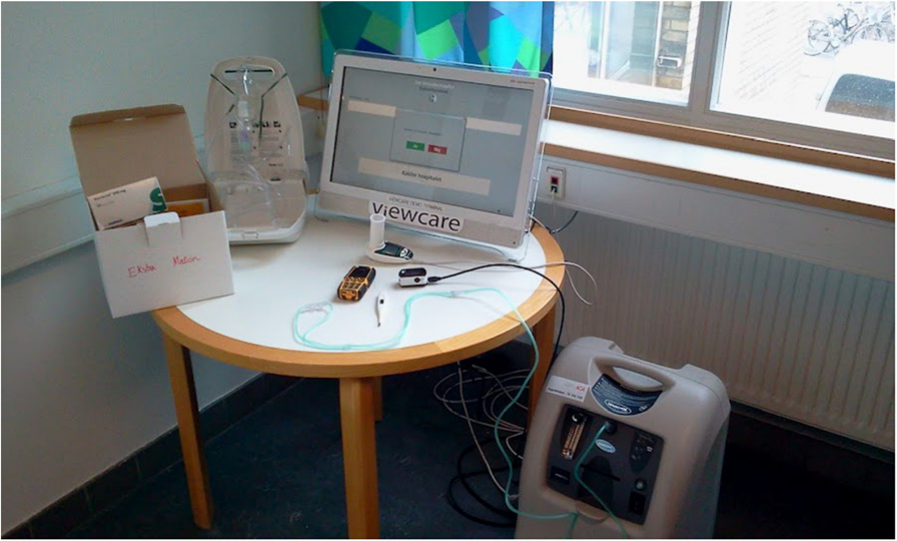
**Photo of the telemedicine technology (TT) equipment used in the Virtual Hospital Trial (VHT).** The photo illustrates the TT equipment used in the patients’ homes and consisted of the following items shown in the photo from left to right: The medicine box containing antibiotics, inhalation medicine, corticosteroids and sedatives; the electronic nebulizer used for inhalation medicine; the touch screen with a built-in webcam; the oxygen compressor on the far right standing on the floor;. the spirometer, pulse oximeter, and thermometer in front of the touch screen were used to send physiological measurements every day to the hospital. Also, the patient phone, which is carried by the nurse working in the acute emergency ward, is in front of the touch screen.

All patients in the experimental group were trained for one hour in a secure environment at the hospital in order to make sure the patients were able to use the technology. A room close to the acute emergency ward was set up to resemble the patient home with the installed technology. They received instructions and training from the health personnel in use of seven items, following a checklist to ensure they were able to use and handle the telemedicine technology (TT) equipment before departure (see list of items´section). The equipment consisted of a videoconference system with a webcam and a touch screen, which allowed the patient direct access to the hospital staff 24 hours a day. Furthermore they were supplied with a pulse oximeter, a spirometer, a thermometer, a nebulizer for aerosolized beta_2_ agonist inhalation, an oxygen compressor and a medicine box containing antibiotics (oral), prednisone (oral), sedative (oral) and beta_2_ agonists (inhalation) There was a USB connection from each monitoring device connecting them to the personal computer (PC) touch screen and data was transmitted via wireless broadband technology (Figure 1). All patients received all the equipment, even patients with reasonable oxygen saturations had to know how to operate the oxygen compressor in case they would need oxygen later on. Most patients were familiar with the spirometer, pulse oximeter, the oxygen supply and medicine from earlier visits to the hospital and from ambulatory visits so the most unfamiliar thing for the patients to use was the touch screen. However, since it only had one possible option (a ‘call hospital’ button on the touch screen) 1 hour of training was judged sufficient before departure.

##### 

**List of items** Checklist and training program for the TT group

1. Patient can use the “call hospital” button on the finger touch screen

2. Patient can perform an oxygen saturation and pulse measurement on the finger tip

3. Patient can perform a lung function measurement

4. Patient can use the oxygen supply

5. Patient can use the nebulizer and corresponding medicine

6. Patient can perform an “acute” call to the hospital

7. Know when to dial the Danish emergency number 112

Patients in the telemedicine technology group (TT group) were transported to their own home within a maximum of 3 to 4 hours after randomization. Upon arrival at home, all patients were asked to redo the seven-item checklist to ensure that the patients still were able to perform the necessary procedures of self-management using the home-monitoring devices, the medication and the technology. The patients were accompanied home by a nurse or a doctor together with a technician who installed the technology and the equipment, a procedure that normally would last half an hour. All patients kept the equipment until considered ready for discharge, and the equipment was physically removed immediately thereafter.

Scheduled contacts with the hospital staff were agreed upon before leaving so that the patient was prepared for daily ward rounds using the video screen at appointed hours. Additional ward rounds and checkups were only planned if judged necessary by the attending doctor. Unscheduled and acute contacts could always be effectuated by the patient pressing the ‘call hospital’ button on the touch screen, a close resemblance to the hospital bed where the patient has a red string to pull when in need of assistance from a nurse. When a patient activated the ‘call hospital’ button a phone carried by the nurse on call would immediately ring, and the nurse could then choose to either talk directly to the patient on the phone or to use a video conference system to contact the patient visually.

If the patient experienced technical problems with the equipment the patient was instructed to call a direct phone number instead or to dial the Danish emergency number 112.

#### ***Control group***

The medical treatment in both groups followed a strict standardized treatment program for COPD patients admitted due to an exacerbation (see list of standard treatment´s section).

##### 

**List of standard treatment** Standard medical treatment regime given in the two groups

• Corticosteroids (oral) Prednisolone 37.5 mg × 1 for 10 days

• Antibiotics (oral) amoxicillin and clavulan acid 500 mg × 3 for 7 days

• Beta-2 agonists and anticholinergics (inhalation) fenoterol and ipratropium bromide via nebulizer in specific intervals

• Oxygen therapy (nasal) when needed

• Sedative (oral) levomepromazine 5 mg × 1 only if needed

The patients allocated to the control group were informed that they would be hospitalized as usual, receiving the conventional standard hospital treatment for an exacerbation.

### Discharge criteria

Prior to the trial 20 Danish lung specialists were asked to write down criteria for discharging a patient with an exacerbation of COPD, and the five most popular criteria were then used in this trial as discharge criteria. The discharge criteria are not applied systematically in usual care in Danish hospitals, and some of the criteria may have delayed discharges that might otherwise have happened outside the trial situation.

Patients in both groups were considered ready for discharge by the attending doctor if they fulfilled the following five criteria: 1) slept >4 hours without awakening from respiratory symptoms, 2) FEV1 not decreasing, 3) clinically stable, 4) condition improved during admission, and 5) oxygen saturation >90 without oxygen or with the regular oxygen supply if they were long-term oxygen users.

### Implementation and organization of telemedicine technology at the acute emergency ward

For the first 9 months of inclusion it was the trial physicians and nurses from the telemedicine research unit at Frederiksberg Hospital who took care of the telemedical patients and operated the 24/7 ‘call center’. The research staff either stayed at the hospital during nights or contacted the patients from their own homes using a PC with the TT solution embedded. Later on, twenty experienced nurses working at the acute emergency department at Frederiksberg University Hospital were trained 4 hours each in operating the TT solution to take over the calls during evenings and nights. During the daytime the research unit remained on call. Nursing staff from the emergency department were instructed to treat the telemedical patients exactly the same way as they would treat them if they had been present at the hospital except from the physical contact, which of course was not possible.

Patients admitted to their own home were represented with a special color on the electronic patient boards in the acute emergency ward indicating they were not physically present in the acute emergency wards but that they were still admitted or ‘outmitted’, which became an everyday expression for a patient treated at home. At the organizational level, the bed in which the patient should have been placed in was kept empty to reflect a decrease in workload at the emergency ward having patients ‘outmitted.’ This was very meaningful in that the emergency staff did not feel as if further workload was being introduced as a result of new treatment modalities.

### Sample size

The trial was originally planned to be conducted as a trial of non-inferiority, which determined the method for calculating sample size based on a prior study where the estimated readmission rate was set to be 23.1% [13,14].

The sample size calculation assuming non-inferiority with a predefined margin of 20% or less, with 5% alpha-error and 80% power, showed that a patient number of 70 in each group was needed. The inclusion number was therefore originally set to be 175 patients with an expected drop out of 20%. However, the required sample size was not attainable in the planned study duration and inclusion was stopped as planned after 18 months. This meant that the study would not reach sufficient power, but reporting of the sample size determination will still be done according to the consort statement recommendation [15].

### Collection of data

All patient data were collected in a case record form (CRF) at baseline, during the intervention, at discharge and again at follow-ups 1, 3 and 6 months after discharge.

Data concerning physiological parameters (pulse, temperature, oxygen saturation, FEV1, and FVC) were collected by a project nurse or project physician or by the ordinary staff at the two hospitals during the ward rounds.

Data concerning readmissions, emergency room visits, rehabilitation, ambulatory visits, deaths, additional prescriptions of prednisone or antibiotics during hospitalization, and length of hospital stays were obtained by searching the hospital databases, The Danish National Patient Registry, and the Danish Civil Registration System, and also by asking the patients at the three follow-up visits. The only data that were solely self-reported from the patient and not obtainable in the hospital databases were the health-related quality of life questionnaires and if there had been any visits to the general practitioner due to COPD. The validated health-related quality of life questionnaires used were: the Saint George Respiratory Questionnaire, (SGRQ), the Clinical COPD questionnaire (CCQ), and the EQ-5D questionnaire, a standardized instrument developed by the Euroqol group.

Also, a user satisfaction questionnaire was handed out to patients in the TT group at discharge and to the nurses who engaged with the ‘outmitted’ patients using the telemedical equipment at the hospital.

The patient user satisfaction questionnaire consisted of 29 questions. Of these, 24 questions were presented in a 5-point Likert-scale (1 strongly disagree through 5 strongly agree) and 5 close-ended questions (yes/no). The questionnaire covers the patient experience in different settings such as the daily ward round, acute calls, the equipment use, and the patient’s general experience of the at home admission with telemedicine technology.

The cognitive status of each patient was evaluated in a substudy using a neuropsychological test battery, including verbal learning, memory capacity and attention and was also evaluated by a self-reported questionnaire: the Subjective Cognitive Functioning (SCF). In the substudy, the patient’s functional status was evaluated by the Instrumental Activity of daily living (IADL)questionnaire.

Another substudy evaluated the patients’ self-efficacy. Self-efficacy was measured using ‘The COPD self-efficacy scale’, which has been validated in a Danish version [16].

### Statistics

All analyses will be done using SAS software version 9.3 http://support.sas.com/software/93. Time to readmission after discharge due to an exacerbation will be assessed using a stratified Cox regression to relax the assumptions of proportional hazards. Differences in readmission rate over time will be assessed using multilevel mixed-effects linear regression analysis with unstructured variance matrix where the baseline values of the outcomes are used as covariates. *P* values below 0.05 will be considered significant for the primary outcome, and *P* values below 0.01 will be considered significant for secondary outcomes. Skewed variables will be power transformed if possible; otherwise, the variables will be transformed into dichotomous responses. Missing values will be replaced using multiple imputations [17]. Survival times will be plotted in a Kaplan-Meier survival curve. Differences between the two groups in prescription of additional antibiotics or steroids due to further exacerbation, need of non-invasive ventilation or mechanical ventilation, emergency room visits, visits to the general practitioner, lung function, bed days, and adverse events will be analyzed with an unpaired Student t-test if data are normally distributed or Mann-Whitney test if not. All categorical data will be analyzed with Chi-square test or Fishers exact test as appropriate. For the health-related quality of life scores, differences from baseline, both within and between study groups, and 95% confidence intervals will be calculated. All analyses will be done on the principle of intention to treat.

### Adverse events

All adverse events and adverse device events that occurred during the 30-day study observation period will be reported in the final paper. An adverse event is defined as any unwanted event happening to the patient during the clinical trial while using the medical equipment or after using the equipment regardless of whether there is a connection between the use of the equipment and the unwanted event or not. A serious adverse event was defined as an event that leads to death, to life threatening damage or disease, to permanent damage to the body, to permanent disability, or to surgical or medical treatment to avoid these serious events. Also events leading to hospital admission or prolongation of the hospital stay were considered serious events. The formal stopping rule was that the study would be stopped after 18 months of inclusion or before if any serious situations occurred that might put a patient’s welfare at risk. This study is a clinical trial concerning telemedical equipment and all adverse events that may or may not be related to the use of the telemedical equipment will be reported in the CRF and in the final report.

### Ethical considerations

The study was approved by the Danish Regional Committee on Scientific Ethics (protocol number H-2-2010-021) and the Danish Data Protection Agency (journal number 2010-41-4684). The trial is registered on clinicaltrials.gov (NCT01155856) and was conducted in accordance with the principles of GCP and The Helsinki Declaration. All participants signed written informed consent forms before randomization. By signing the consent form the patient accepted that technicians from the IT company, Viewcare, would install the telemedical equipment in their home and would remove it again at discharge. Patients also accepted having the equipment turned on at all times so that it was always possible to make contact. Patients in the control group were asked to fill out questionnaires and had three follow-up visits, but otherwise had no inconveniences in connection to their participation in the trial.

## Results

From June 2010 to December 2011, 57 patients were randomized. Average monthly recruitment for the trial was 3,2 patients. Follow-up visits ended in May 2012.

## Discussion

In Denmark, 28.5% COPD patients were readmitted within 1 month after discharge [18]. The study design for the VHT was directed at selecting the population of COPD patients who are most frequently hospitalized and would potentially benefit from the TT solution in terms of reduced risk of readmissions, emergency room visits, increased health-related quality of life etcetera The design and methods of the VHT differs from previous trials investigating telemedicine solutions for COPD patients. First, only COPD patients with an acute need of hospital treatment were included. Secondly, the patients received comparable medical treatment and attention in both groups with the only difference being the location of the patient, which made it a true ‘telemedical’ intervention with no physical contact with the health personnel. Thirdly, pre-specified discharge criteria were necessary to make the discharges in both groups as unbiased and objective as possible. Fourthly, this trial was based on a telemedical intervention that was actually embedded in the routine clinical care by staff nurses not otherwise involved in the study at the hospital, and the results may have implications on future use and implementation of telemedicine in the hospital sector. Twenty acute emergency ward nurses were trained to deal with telemedical patients from two hospitals, and these patients became part of their daily routine. Their experiences with the TT solution may be valuable for other researchers in the field of organizational research since there is lack of reporting in the literature for implementation of telemedicine interventions in the daily routine care [7].

When it comes to the internal validity of the study, we believe we have taken several initiatives to minimize bias by using consecutive enrollment and central randomization. Due to the nature of the trial it has not been possible to blind patients and healthcare personnel, which introduces the risk of performance, attrition and assessment bias. However, risk of bias may be high for some outcomes and low for others. For example, knowledge of the assigned intervention may affect behavioral outcomes (such as number of hospital visits), while not affecting physiological outcomes or mortality. Thus, assessments of risk of bias resulting from lack of blinding may need to be made separately for different outcomes. However, unblinded studies can take other measures to reduce the risk of bias, such as treating patients according to a strict protocol to reduce the risk of differential behaviors by patients and healthcare providers. We believe we have tried to reduce the risk by treating the patients according to a very strict medical protocol and by discharging them according to the same discharge criteria.

The validity of administrative data such as number of admissions, emergency visits, discharge, etcetera is very high in Denmark, due to The Danish National Patient Registry, which has collected nationwide data on all somatic hospital admissions since 1977 and on all outpatients and emergency patients since 1995.

## Conclusions

The VHT is to our knowledge the first trial to investigate an at-home admission supported by TT compared with a conventional admission due to an exacerbation of COPD. We believe it is the first randomized trial to describe a method where the telemedicine solution was actually implemented in the daily routine clinical care at an acute emergency hospital ward during evening and nights.

The method developed in this study allowed for one hospital to take over the care of COPD patients from another hospital and potentially for even more hospitals. This is the first step of a potential re-organization of hospital healthcare services using telemedicine to connect to other hospitals creating new collaborations and new offers for the patients.

The results of the trial may have implications for future hospital treatment options, as patients can be equipped with telemedicine solutions to avoid expensive hospital admittances when their disease deteriorates. Further, this study is an example of a health service transformation mediated by welfare technology.

## Abbreviations

CCQ: The clinical COPD questionnaire; COPD: chronic obstructive pulmonary disease; CRF: case record form; ECG: electrocardiogram; EQ-5D: self-report questionnaire designed by the Euroqol group to measure health status; FEV1: forced expiratory volume in 1 second; FEV1%: forced expiratory volume in 1 second in percent of expected value; FVC: forced vital capacity; GOLD: the global initiative for chronic obstructive lung disease; IADL: instrumental activity of daily living; NIV: non-invasive ventilation; PC: personal computer; PC02: carbon dioxide partial pressure in arterial blood; RCT: randomized controlled trial; SCF: Subjective Cognitive Functioning questionnaire; SGRQ: St. George’s respiratory questionnaire; TT: telemedicine technology; USB: universal serial bus; VHT: The Virtual Hospital Trial; WHO: World Health Organization.

## Competing interests

None of the authors and none of the relatives of the authors have a relation to, or income from, the telemedicine industry. Klaus Phanareth is head and chairman of the Danish Society for Clinical Telemedicine but did not receive any payment whatsoever for this post, and the society is independent of the telemedicine industry.

## Authors’ contributions

KP developed the design and study protocol. KP, ASJ and LCL collected data. ASJ drafted the manuscript. LCL, BØ, SRH and KP critically revised the manuscript, and all authors read and approved the final version.

## Authors’ information

ASJ is an MD and PhD student at Bispebjerg & Frederiksberg University Hospital, Denmark. LCL is an MD at Herlev University Hospital, Medical Department O, Denmark. BØ is an Associate Professor at The Research Unit of Nursing, Institute of Clinical Research, University of Southern Denmark. SRH is the Head of Research, a PhD and an MSN at The Research Unit of Clinical Nursing, Bispebjerg & Frederiksberg University Hospital, Denmark. KP is an MD, a PhD and Head of Telemedicine Research at Frederiksberg University Hospital, Denmark.
